# *Vibrio cholerae* interactions with *Mytilus galloprovincialis* hemocytes mediated by serum components

**DOI:** 10.3389/fmicb.2013.00371

**Published:** 2013-12-09

**Authors:** Laura Canesi, Elisabetta Pezzati, Monica Stauder, Chiara Grande, Margherita Bavestrello, Adele Papetti, Luigi Vezzulli, Carla Pruzzo

**Affiliations:** ^1^Dipartimento di Scienze della Terra, dell’Ambiente e della Vita, Università di GenovaGenova, Italy; ^2^Dipartimento di Scienze del Farmaco, Università di PaviaPavia, Italy

**Keywords:** *Vibrio cholerae*, adherence, mussel, hemocytes, serum

## Abstract

Edible bivalves (e.g., mussels, oysters) can accumulate large amount of bacteria in their tissues and act as passive carriers of pathogens to humans. Bacterial persistence inside bivalves depends, at least in part, on hemolymph anti-bacterial activity that is exerted by both serum soluble factors and phagocytic cells (i.e., the hemocytes). It was previously shown that *Mytilus galloprovincialis* hemolymph serum contains opsonins that mediate D-mannose-sensitive interactions between hemocytes and *Vibrio cholerae* O1 El Tor bacteria that carry the mannose-sensitive hemagglutinin (MSHA). These opsonins enhance phagocytosis and killing of vibrios by facilitating their binding to hemocytes. Since *V. cholerae* strains not carrying the MSHA ligand (O1 classical, non-O1/O139) are present in coastal water and can be entrapped by mussels, we studied whether in mussel serum, in addition to opsonins directed toward MSHA, other components can mediate opsonization of these bacteria. By comparing interactions of O1 classical and non-O1/O139 strains with hemocytes in artificial sea water and serum, it was found that *M. galloprovincialis* serum contains components that increase by at approximately twofold their adhesion to, association with, and killing by hemocytes. Experiments conducted with high and low molecular mass fractions obtained by serum ultrafiltration indicated that these compounds have molecular mass higher than 5000 Da. Serum exposure to high temperature (80°C) abolished its opsonizing capability suggesting that the involved serum active components are of protein nature. Further studies are needed to define the chemical properties and specificity of both the involved bacterial ligands and hemolymph opsonins. This information will be central not only to better understand *V. cholerae* ecology, but also to improve current bivalve depuration practices and properly protect human health.

## INTRODUCTION

Microorganisms in seawater can be entrapped by filter feeding invertebrates that sieve suspended particles from the aquatic environment (Vazquez-Novelle et al., 2005; Beleneva et al., 2007). Accumulation of bacteria pathogenic to humans in the tissues of edible bivalves is of great concern to public health; in fact, consumption of raw or inadequately cooked bivalves has been implicated in numerous food poisoning outbreaks (Suzita et al., 2009; Scallan et al., 2011). Bivalve microbiological depuration (purification) in controlled waters is used worldwide to reduce the number of unwanted microorganisms to acceptable levels for human consumption (Teplitski et al., 2009). However, bacteria show different sensibility to depuration treatment; for instance, some *Vibrio* species have been reported to be resistant to the process and are able to persist and multiply within shellfish tissues (Tamplin and Capers, 1992; Murphree and Tamplin, 1995).

A relationship between microbial resistance to depuration and sensitivity to hemolymph bactericidal activity has been suggested (Harris-Young et al., 1995; Canesi et al., 2002, 2005). Shellfish hemolymph contains both hemocytes, which are responsible for cellular defense mechanisms (i.e., phagocytosis, production of reactive oxygen intermediates, and release of lysosomal enzymes), and soluble factors (e.g., opsonizing lectins and hydrolytic enzymes; Canesi et al., 2002; Pruzzo et al., 2005a). The capacities of different bacteria to survive hemolymph microbicidal activity depend on their sensitivities to combinations of these factors (Duperthuy et al., 2011; Balbi et al., 2013). Elucidation of underlying molecular mechanisms is crucial to improve current depuration practices and properly protect human health.

*Vibrio cholerae* is part of the endogenous bacterial component in estuarine areas (Kaper et al., 1995; Lipp et al., 2002). Only two serogroups, O1 (classical and El Tor biotypes) and O139 have been associated with cholera epidemic disease although nearly 200 serogroups of *V. cholerae* (named “non-O1/O139”) have been described (Kaper et al., 1995; Nelson et al., 2009). The great majority of non-O1/O139 strains do not produce cholera toxin and are not associated with epidemic diarrhea. These strains are much more commonly isolated from the environment than are O1/O139 strains, and are occasionally isolated from cases of diarrhea usually due to consumption of raw or partially cooked shellfish (Iwamoto et al., 2010; Oliver et al., 2013).

Recent data on surface components involved in interactions of *V. cholerae* O1 with hemocytes of *Mytilus galloprovincialis,* an economically important, and appreciated seafood in the Mediterranean area, indicated that hemolymph serum contains opsonins specifically directed toward the “mannose-sensitive hemagglutinin (MSHA)” (Zampini et al., 2003), an adhesin expressed by El Tor and O139 strains (other *V. cholerae* strains either do not carry the *msha* gene or do not express it, i.e., strains of O1 serogroup, classical biotype; Chiavelli et al., 2001). It was suggested that D-mannose containing serum opsonins, capable to specifically react with MSHA, are involved in *V. cholerae* El Tor phagocytosis, and killing by mussel hemocytes (Zampini et al., 2003).

However, the above data did not rule out the possibility that in mussel serum, in addition to opsonins directed toward MSHA, other components are present that mediate interactions with hemocytes of vibrios different from O1 El Tor. To explore this possibility, we tested the capability of *M. galloprovincialis* serum to promote phagocytosis and killing by hemocytes of *V. cholerae* bacteria of O1 classical biotype and non-O1/O139 serogroups. The obtained results indicate that opsonizing molecules directed toward these bacteria are actually present in *M. galloprovincialis* hemolymph serum; a preliminary analysis of these components and the involved bacterial antigens is presented.

## MATERIALS AND METHODS

### BACTERIA AND CULTURE CONDITIONS

*Vibrio cholerae* CD81 (O1 classical ) was kindly provided by Dr. B. S. Srivastava (Microbiology Division, Central Drug Research Institute, Lucknow 226001, India); RC60, RC66, and RC69 (non-O1/O139) were kindly provided by Dr. A. Huq (Maryland Pathogen Research Institute, University of Maryland, College Park, MD 20742, USA). N16961 ATCC^®^ 39315^TM^ (O1 El Tor) was also used in some experiments. All cultures were grown in Luria-Bertani (LB) broth or agar under static conditions at 37°C. To radiolabel bacteria, strains were grown overnight in LB broth containing 10 μCi of [methyl-^3^H]thymidine (25 Ci/mmol) ml^-^^1^, were then harvested by centrifugation (3,000 × *g* for 15 min at 4°C), washed three times with phosphate-buffered saline (PBS; 0.1 M KH_2_PO_4_, 0.1 M Na_2_HPO_4_, 0.15 M NaCl, pH 7.2–7.4), and resuspended in PBS at an A_650_ of 1(2 × 10^8^ to 4 × 10^8^ bacteria ml^-^^1^). The number of counts per minute (cpm) per milliliter and the number of bacteria per milliliter were evaluated in triplicate samples to calculate the efficiency of cell labeling (number of bacteria per cpm) that varied in different bacterial preparations from 180 to 350. Artificial sea water [ASW; 35‰ (wt/vol) salinity, pH 7.9], filtered onto 0.22 μm-pore-size Millipore filters (Bedford, MA, USA), was used throughout the experiments.

### PREPARATION OF MUSSELS HEMOCYTE MONOLAYERS

Mussels (*M. galloprovincialis* Lam.) were obtained from the depuration plant Casa del Pescatore (Cattolica, Italy). Animals were transferred to the laboratory, cleaned of epibiota and kept in an aquarium at 16°C in static tanks containing ASW (1 l/animal) for 1–3 days before use; sea water was changed daily. Hemolymph was extracted from the posterior adductor muscle of the mussels by using a sterile 1 ml syringe with a 18-gauge, 0.5-in. long needle. After the needle was removed, the hemolymph was filtered through sterile gauze and pooled. To prepare hemocyte monolayers, an approximately 0.3-ml portion of hemolymph (corresponding to about 2 × 10^6^ to 3 × 10^6^ cells) was seeded onto glass coverslips (20 by 22 mm) placed in plastic culture dishes. The coverslips were incubated at 18°C for 30 min. Non-adherent hemocytes were removed by gently washing the preparations three times with 3 ml of ASW (Zampini et al., 2003).

To obtain hemolymph serum (i.e*.*, hemolymph free of cells), the whole hemolymph was centrifuged at 50 × *g* for 10 min; the supernatant was then passed through a filter (pore size, 0.22 μm).

### ADHESION TO AND ASSOCIATION WITH HEMOCYTE MONOLAYERS

Evaluation of bacterial adherence to hemocytes was performed as follow: aliquots (1.5 ml) of either ASW or hemolymph serum containing radiolabeled bacteria at a final concentration of 2 × 10^7^ to 3 × 10^7^ bacteria ml^-^^1^ were added to monolayers, and the dishes were incubated with gentle shaking at either 4°C (to evaluate adhering bacteria) or 18°C (to evaluate associated bacteria = adhering + internalized). Triplicate preparations were made for each sample. After 60 min incubation, the cover slips were rinsed with cold ASW, and transferred to PICO-FLUOR^TM^15 scintillation fluid (Packard Instruments Company Inc., Meriden, CT, USA). For each sample, the number of bacteria per monolayer was calculated using the efficiency of cell labeling. Background counts due to bacterial attachment to cover-slips were also evaluated (typically 50–250 cpm) and subtracted from the sample values. In experiments performed to define the chemical nature of the bacterial component(s) involved in interactions with hemocytes, bacteria were pretreated with pronase E and sodium meta-periodate. Pronase E was added to bacterial suspensions at a final concentration of 100 pgml^-^^1^. The suspensions were then incubated for 1 h at 37°C in a shaking water bath and centrifuged. The pellets were resuspended in PBS to the original volume. Sodium meta-periodate pretreatment of bacteria was performed in PBS containing sodium meta-periodate at a final concentration of 1 mM. The suspensions were incubated at room temperature for 10 min, washed twice and resuspended in PBS.

### BACTERIAL KILLING BY HEMOCYTES

To evaluate bacterial sensitivity to killing by hemocytes, *V. cholerae* suspensions (about 10^7^ bacteria ml^-^^1^) were added to hemocyte monolayers at 18°C in the presence of hemolymph serum as described above. Triplicate preparations were made for each sampling time. Immediately after the inoculum (*T* = 0) and after 60 min of incubation at 18°C supernatants were collected from monolayers, and hemocytes were lysed by adding 5 ml of cold distilled water and by 10 min of agitation. The collected monolayer supernatants and hemocyte lysates were pooled and 10-fold serially diluted in PBS; aliquots (100 μl) of the diluted samples were plated onto LB agar, and after overnight incubation at 37°C the number of colony-forming units (CFU) per milliliter in the hemocyte monolayer (representing culturable bacteria survived to hemocyte bactericidal activity) was determined. Percentages of killing at 60 min were then determined relative to values obtained at *T* = 0. To evaluate the presence of endogenous bacteria in hemocytes, controls were performed with hemocyte monolayers without bacteria. The number of CFU in controls never exceeded 0.1% of those enumerated in experimental samples. To detect and correct for bacterial growth in hemolymph serum, separate samples were seeded with bacteria and 1.5 ml of sterile hemolymph serum. No appreciable bacterial growth was observed at the same time intervals used in killing experiments.

### SERUM FRACTIONATION

Serum was fractionated into low and high molecular mass fractions (LMM and HMM) by ultrafiltration using the Vivaflow 200 complete system (Vivascience AG, Feodor-Lynen-Strasse 21, 30625 Hannover, Germany) comprising a pump (240 V), tubing, 500 ml sample/diafiltration reservoir, and a membrane 5,000 MWCO PES for ultradiafiltration (Vivascience). The LMM 5000 cutoff was chosen since electrophoretic separation of mussel soluble serum proteins (in both 1D and also 2D gels used for proteomic studies in different conditions) generally yields protein bands with MM generally ≥10,000. A diafiltrate, i.e., a LMM containing all the compounds with molecular masses less than 5000 Da, and a retentate, i.e., a HMM containing all the compounds with molecular masses greater than 5000 Da were obtained and, after restoring the initial volume, were tested.

### STATISTICS

Experiments were repeated at least three times. Data shown in the Figures are the mean values ± standard deviation obtained in one representative experiment performed in triplicate. Data were analyzed for significance by the Mann–Whitney *U* test. Differences were considered significant at *P* < 0.05.

## RESULTS

### *IN VITRO* INTERACTIONS OF *Vibrio* STRAINS WITH HEMOCYTES AND SENSITIVITY TO KILLING

**FIGURE 1:**
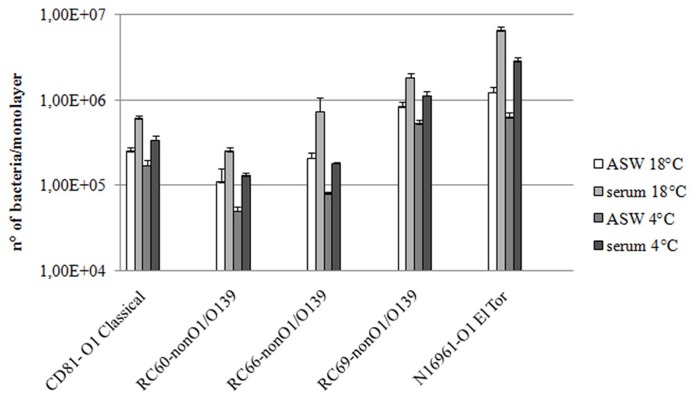
***Vibrio cholerae* strains association to (18°C) and adhesion with (4°C) hemocyte monolayers**. All the experiments were performed in the presence and in the absence of hemolymph serum, after 60 min of incubation. *P* < 0.05.

We studied interactions with hemocytes of *V. cholerae* strains that either do not carry the *msha* gene (RC60, RC66, and RC69 [non-O1/O139 serogroups]) or do not express it (CD81 [O1 serogroup, classical biotype]). Bacteria were added to hemocyte monolayers in both ASW and hemolymph serum at 18°C. At this temperature, the number of associated (adhering + internalized) bacteria was evaluated. **Figure 1** shows that in hemolymph serum the association efficiency of the tested strains was 2.2–3.5-fold higher than in ASW (*P* ≤ 0.05).

To clarify to what extent the observed differences in association were due to different adhesion efficiencies, the same assays were performed at 4°C, a condition that almost completely inhibits the internalization process (Zampini et al., 2003; **Figure 1**). The presence of hemolymph serum increased the number of vibrios adhering to hemocytes by 2.1–2.6-fold in comparison to ASW (*P* ≤ 0.05; **Figure 1**). Moreover, as expected, at 4°C the number of bacteria interacting with hemocytes was lower than that at 18°C. Interestingly, the increase in both association with and adherence to mussel hemocytes of the tested strains was about twofold lower than that observed with the MSHA-positive *V. cholera*e N16961 strain, used for comparison.

**FIGURE 2:**
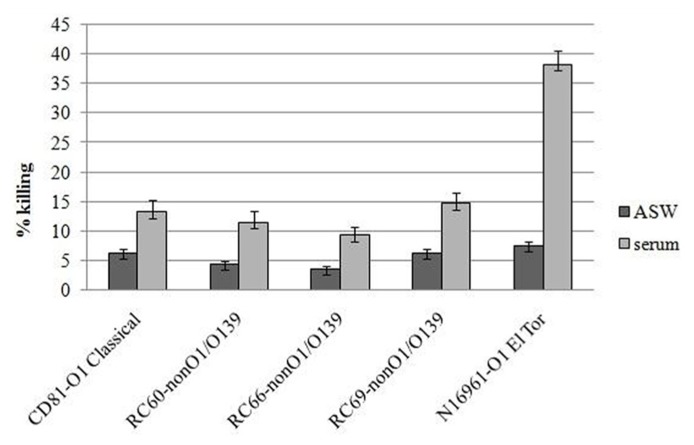
***Vibrio cholerae* strains killing by hemocyte monolayers**. All the experiments were performed in the presence and in the absence of hemolymph serum, after 60 min of incubation. *P* < 0.05.

*Vibrio cholerae* strains were then tested for their ability to resist to killing by hemocytes, both in the presence and in the absence of serum (**Figure 2**). It was found that, after 60 min incubation, the percentage of killed bacteria compared to that at *T* = 0 ranged from 9.3 to 14.7% in the experiments performed in serum and from 3.5 to 6.3% in experiments performed in ASW. Differences between the two experimental conditions were statistically significant (*P* ≤ 0.05). As for association and adhesion, the serum-mediated increase in killing efficiency of the tested strains by hemocytes was about twofold lower than that observed with *V. cholera*e N16961 strain carrying the MSHA.

As a whole, these results indicate that mussel serum, in addition to opsonins directed toward MSHA, contains factors that mediate binding between *Vibrio* cell wall component(s) and hemocyte surface receptor(s), and promote internalization and killing of these bacteria.

### PRELIMINARY ANALYSIS OF BACTERIAL SURFACE LIGANDS AND SERUM COMPONENTS INVOLVED IN INTERACTIONS OF *Vibrio* STRAINS WITH HEMOCYTES

**FIGURE 3:**
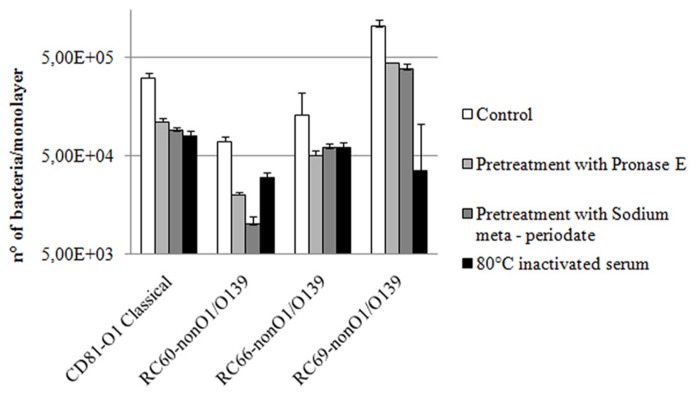
**Effect of bacterial pre-treatment with pronase E or sodium meta-periodate** and serum heat inactivation on *V. cholerae* strains adhesion to hemocyte monolayers (4°C) in serum, after 60 min of incubation.** *P* < 0.05.

In a first attempt to define the chemical nature of bacterial surface components involved in *V. cholerae* interactions with hemolymph, adhesion to hemocytes of the tested strains in the presence of serum was evaluated after bacteria had been treated with either pronase E or sodium meta-periodate, which oxidizes polysaccharides. As shown in **Figure 3**, both treatments caused a decrease in serum-mediated attachment to hemocytes. In comparison to untreated controls, pronase E and sodium meta-periodate treatments reduced the interactions by 2.3–3.5 and 2.7–7.0-fold, respectively. Meta-periodate oxidation and pronase E digestion did not affect bacterial viability, as demonstrated by viable counts (data not shown).

To preliminarily assess the main chemical properties of serum factors capable to promote *Vibrio* strain interactions with hemocytes, hemolymph serum was first exposed (15 min) to different temperatures, ranging from 45 to 80°C, then used in experiments to evaluate efficiency of bacterial adhesion to hemocytes. Serum progressively lost its capability to promote *V. cholerae* attachment to hemocytes with increasing temperature from 45 to 80°C. In particular, the number of bacteria adhering to hemocytes in the presence of serum incubated at 80°C was 2.2–3.9-fold lower than that observed in the presence of untreated serum (**Figure 3**), and similar to those obtained in experiments performed in ASW (**Figure 1**).

**FIGURE 4:**
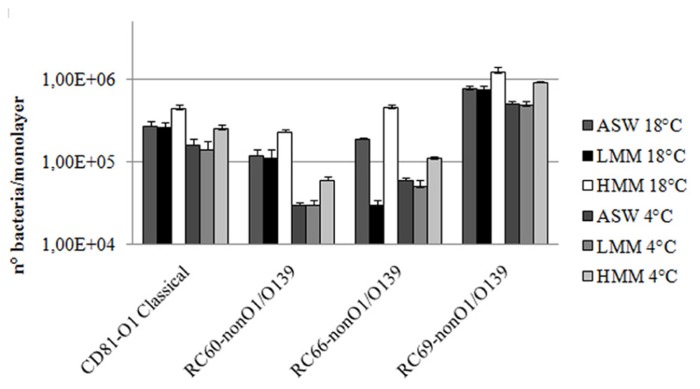
***Vibrio cholerae* strains association to (18°C) and adhesion with (4°C) hemocyte monolayers in the presence of hemolymph serum LMM and HMM fractions**. *P* < 0.05.

Serum was then fractionated into LMM and HMM fractions by ultrafiltration (see Methods). LMM fraction contained all the compounds with molecular masses lower than 5000 Da, and HMM contained all the compounds with molecular masses greater than 5000 Da. Capability of both fractions to promote adhesion to and association with hemocytes of the tested bacteria was analyzed. As shown in **Figure 4**, whereas LMM fraction did not cause any statistically significant increase in bacterial interactions with hemocytes, the HMM fraction caused a 1.6–2.4-fold increase of both adhesion to and association with monolayers of the tested strains. When the experiments were performed using a mixture of both LMM and HMM fractions, results similar to those obtained with HMM alone were obtained (not shown).

## DISCUSSION

We previously showed that *M. galloprovincialis* hemolymph serum contains soluble factors that are involved in D-mannose-sensitive interactions between hemocytes and *V. cholerae* O1 El Tor carrying the MSHA adhesin (Zampini et al., 2003). These opsonins enhance phagocytosis and killing of these bacteria by facilitating their binding to hemocytes.

However, other *V. cholerae* strains, including human epidemic clones (O1 classical), not carrying the MSHA ligand are present in coastal waters (Pruzzo et al., 2005b; Oliver et al., 2013) and can be entrapped by mussels. These vibrios, which can persist and replicate in bivalves, were selected for the current study to evaluate if additional serum factors, different from those directed toward MSHA, can affect *V. cholerae* adhesion to hemocytes and sensitivity to killing, modulating its densities inside mussels.

By comparing interactions of O1 classical and non-O1/O139 strains with hemocytes in ASW and serum, it was found that *M. galloprovincialis* serum actually contains components that promote adhesion to, association with and killing by hemocytes of bacteria that do not carry the MSHA. Serum components different from those acting toward MSHA are likely to be involved as suggested by the fact that serum-mediated increase in bacterial interactions with hemocytes is higher toward *V. cholerae* O1 El Tor (carrying MSHA) than toward the other tested vibrios (without MSHA). Interestingly, when comparing efficiencies of interactions with hemocytes and killing sensitivity of the different tested strains, a variability among MSHA-negative isolates was observed. This might be due, at least in part, to differences in bacterial surface hydrophobicity and/or surface charge. Moreover, the most adhesive strains might carry surface molecules mediating non-opsonic interactions with hemocyte membrane.

The preliminary analysis of the chemical nature of the involved bacterial ligand(s) indicated that both cell wall protein(s) and carbohydrate(s) are responsible for the interactions of non-El Tor *V. cholerae* strains with serum. In fact, using vibrios pre-treated with proteinase E or sodium meta-periodate that disrupts cell surface polysaccharide, the observed serum-mediated increase of adhesion and association showed a significant reduction in comparison to untreated controls. On the other hand, the fact that serum exposure to high temperature (80°C) abolished its capability to promote adhesion to and association with phagocytes suggests that heat-sensitive serum components are involved in the opsonizing activity. Experiments conducted with HMM and LMM fractions obtained by serum ultrafiltration indicate that these compounds have molecular mass higher than 5000 Da.

Further studies are needed to define the chemical nature and specificity of both the involved bacterial adhesins and hemolymph opsonins as well as physical and chemical conditions promoting or inhibiting such interactions inside bivalves *in vivo*. This information will be crucial to better understand the strategies used by mussels to control bacterial diffusion in their tissues and by *V. cholerae* to persist and spread in the aquatic environment.

Consumption of raw or partially cooked bivalves has been implicated in numerous food poisoning outbreaks. Thus, their microbial flora is of great concern to public health (Suzita et al., 2009; Scallan et al., 2011). The ability of bivalves to eliminate pathogens from their tissues is due, at least in part, to the ability of hemocytes to bind, phagocytize, and kill these bacteria (Canesi et al., 2002; Pruzzo et al., 2005a). Deciphering molecular mechanisms affecting pathogenic bacteria – hemocytes interactions represents the basis to improve depuration treatment and to prevent transmission of pathogens to humans through consumption of raw or partially cooked bivalves. As an example, to reduce the load of unwanted bacteria inside bivalves, depuration might be conducted in conditions that favor phagocytosis of pathogens and their clearance from hemolymph.

Unraveling the cellular and molecular mechanisms associated with hemolymph anti-bacterial activity is also central to understand the pathogenesis of bivalve diseases in cultured and wild populations of species susceptible to *Vibrio* spp. infection, and to set up new strategies to control summer mortalities affecting the bivalve production (e.g., *Crassostrea gigas* oysters) in aquaculture worldwide (Gay et al., 2004; Garnier et al., 2007).

## Conflict of Interest Statement

The authors declare that the research was conducted in the absence of any commercial or financial relationships that could be construed as a potential conflict of interest.

## AUTHOR CONTRIBUTIONS

Laura Canesi and Luigi Vezzulli organized and performed most experiments. Chiara Grande and Margherita Bavestrello took care of aspects related to mussel maintenance in aquarium and hemocyte monolayer preparations. Elisabetta Pezzati cultured and controlled bacterial strains. Monica Stauder supplied technical assistance. Adele Papetti carried out serum fractionation. Carla Pruzzo designed the experiments and supervised.
